# Identification and characterization of a core set of ROS wave‐associated transcripts involved in the systemic acquired acclimation response of Arabidopsis to excess light

**DOI:** 10.1111/tpj.14205

**Published:** 2019-01-30

**Authors:** Sara I. Zandalinas, Soham Sengupta, David Burks, Rajeev K. Azad, Ron Mittler

**Affiliations:** ^1^ The Division of Plant Sciences College of Agriculture, Food and Natural Resources University of Missouri School of Medicine Christopher S. Bond Life Sciences Center University of Missouri 1201 Rollins St Columbia MO 65201 USA; ^2^ The Department of Surgery University of Missouri School of Medicine Christopher S. Bond Life Sciences Center University of Missouri 1201 Rollins St Columbia MO 65201 USA; ^3^ Department of Biological Sciences College of Science University of North Texas 1155 Union Circle #305220 Denton TX 76203‐5017 USA; ^4^ Department of Mathematics University of North Texas Denton TX 76203 USA

**Keywords:** transcriptomics, reactive oxygen species (ROS) wave, systemic signaling, systemic acquired acclimation (SAA), MYB30, WRKY, light stress, H_2_O_2_ signaling, *Arabidopsis thaliana*

## Abstract

Systemic acquired acclimation (SAA) plays a key role in optimizing growth and preventing damage associated with fluctuating or abrupt changes in the plant environment. To be effective, SAA has to occur at a rapid rate and depend on rapid signaling pathways that transmit signals from affected tissues to all parts of the plant. Although recent studies have identified several different rapid systemic signaling pathways that could mediate SAA, very little information is known about the extent of their involvement in mediating transcriptomic responses. Here we reveal that the systemic transcriptomic response of plants to excess light stress is extensive in its context and involves an early (2 min) and transient stage of transcript expression that includes thousands of genes. This early response is dependent on the respiratory burst oxidase homolog D protein, and the function of the reactive oxygen species (ROS) wave. We further identify a core set of transcripts associated with the ROS wave and suggest that some of these transcripts are involved in linking ROS with calcium signaling. Priming of a systemic leaf to become acclimated to a particular stress during SAA involves thousands of transcripts that display a rapid and transient expression pattern driven by the ROS wave.

## Significance Statement

The reactive oxygen species (ROS) wave is required for a rapid, early and transient systemic transcriptomic response to light stress that includes thousands of transcripts, many of them important for light stress acclimation. A core set of transcripts that includes several transcriptional regulators involved in H_2_O_2_ signaling is directly associated with the ROS wave and could be associated with systemic responses to many other stresses.

## Introduction

Light stress occurs in plants when the capacity of the plant to harvest light and use it for CO_2_ fixation is overwhelmed by excess light energy. Under such conditions the photosynthetic antennas absorb photons at a rate that is higher than the capacity of the photosynthetic centers to channel electrons through the electron transport chain (ETC) mechanisms, resulting in the production of singlet oxygen and other excited molecules. Electrons flowing through the ETC may additionally be transferred to alternative acceptors such as oxygen, resulting in the formation of superoxide radicals (Asada, 2006; Dietz, 2015; Alric and Johnson, 2017). Because CO_2_ fixation is dependent on stomatal conductance and temperature, excess light may pose an even bigger challenge to plants when it is combined with other stresses, such as drought or temperature stress, that limit the rates of CO_2_ fixation (Mittler, 2006). Because light plays such a pivotal role in the life of photosynthetic organisms, plants have evolved many different acclimation and adaptation mechanisms to counter the effect of excess light stress. These include pathways for adjusting the size of the antenna complexes, different quenching mechanisms, and pathways to scavenge the excess reactive oxygen species (ROS) produced (Asada, 2006; Li *et al*., 2009; Dietz, 2015). These and other pathways are not only triggered at the site of excess light stress, but also at systemic tissues that are not yet subjected to the stress (Karpinski *et al*., 1999; Rossel *et al*., 2007; Kangasjarvi *et al*., 2009; Szechyńska‐Hebda *et al*., 2010, 2017; Gorecka *et al*., 2014; Gilroy *et al*., 2016; Devireddy *et al*., 2018). This phenomenon, termed systemic acquired acclimation (SAA), allows the systemic non‐stressed tissues of the plant to prepare for and acclimate to the impending stress condition(s), and is thought to play a key role in the acclimation of plants to many different abiotic stresses. The different systemic signaling pathways mediating SAA in response to excess light and other stresses in plants include ROS and calcium waves, electric signals, plant hormones such as abscisic acid (ABA) and jasmonic acid (JA), and hydraulic waves (Karpinski *et al*., 1999; Rossel *et al*., 2007; Kangasjarvi *et al*., 2009; Miller *et al*., 2009; Szechyńska‐Hebda *et al*., 2010, 2017; Mittler *et al*., 2011; Christmann *et al*., 2013; Suzuki *et al*., 2013; Gilroy *et al*., 2014, 2016; Gorecka *et al*., 2014; Ciszak *et al*., 2015; Matsuo and Oelmüller, 2015; Carmody *et al*., 2016; Guo *et al*., 2016; Choi *et al*., 2017; Devireddy *et al*., 2018).

Recent transcriptomics and metabolomics studies demonstrated that molecular and metabolic responses to excess light stress can occur within seconds to minutes of light stress initiation (Suzuki *et al*., 2015; Choudhury *et al*., 2018), and that recovery from light stress is accompanied by rapid alterations in transcript stability and abundance (Crisp *et al*., 2017). A recent study also reported that a rapid stomatal response to excess light stress occurs within minutes in local and systemic leaves of *Arabidopsis thaliana*, and that the propagation of the systemic stomatal response from the local leaf to the entire plant canopy is mediated by the ROS wave (Devireddy *et al*., 2018). This response was also dependent on the function of the plant hormone ABA, and slow anion channel‐associated 1 (SLAC1) and guard cell hydrogen peroxide resistant 1 (GHR1) proteins (Devireddy *et al*., 2018).

The ROS wave was initially characterized by Miller *et al*. (2009) and shown to depend on the function of the respiratory burst oxidase homolog D (RBOHD) protein. The ROS wave propagates from the stressed tissue to almost all other parts of the plant via a cell‐to‐cell auto‐propagating process of ROS‐induced ROS production (Mittler *et al*., 2011; Zandalinas and Mittler, 2017). Each cell along the path of the systemic ROS wave signal is triggered therefore to produce ROS in response to sensing of ROS produced by the preceding cell in the path and the enhanced levels of ROS produced during this process accumulate at the apoplast. The ROS wave is coordinated with a systemic calcium wave and each of these two waves requires the function of the other (Evans *et al*., 2016; Toyota *et al*., 2018). Interestingly, the ROS wave was also found to be required for the propagation of a certain type of electric signals (variation potentials) suggesting that the ROS, calcium and electric waves are coordinated in plants (Suzuki *et al*., 2013).

Although the ROS wave was shown to be triggered by light stress (Choudhury *et al*., 2018; Devireddy *et al*., 2018), to be required for the SAA of plants to excess light in Arabidopsis (Suzuki *et al*., 2013), and to be required for the systemic propagation of light stress‐induced systemic stomatal responses (Devireddy *et al*., 2018), very little information is known about the changes in gene expression regulated or coordinated by this signal. In addition, because responses to light stress have been shown to occur much faster than previously anticipated (Suzuki *et al*., 2015; Crisp *et al*., 2017; Choudhury *et al*., 2018; Kollist *et al*., 2018), it is unclear what transcriptomics responses are triggered in systemic and local leaves within minutes of light stress application to a local leaf, and whether or not these responses are dependent on the function of the ROS wave. To address these questions, we conducted transcriptomics time‐course (0, 2, 4 and 8 min) light stress experiments sampling local and systemic leaves of wild type and *rbohD* Arabidopsis plants. In addition, we conducted transcriptomics experiments studying the response of plants to external H_2_O_2_, simulating H_2_O_2_ entry from the apoplast into cells, as well as pharmacology experiments blocking the light stress‐induced ROS wave with diphenyleneiodonium (DPI). Our analysis identified a core set of ROS wave‐associated transcripts involved in the SAA response of Arabidopsis to excess light. We further show that at least six of these genes are required for light stress acclimation, and propose that the transcriptional regulator MYB30 plays a key role in linking the ROS and calcium waves.

## Results

### Local and systemic responses to excess light stress in Arabidopsis

**Figure 1:**
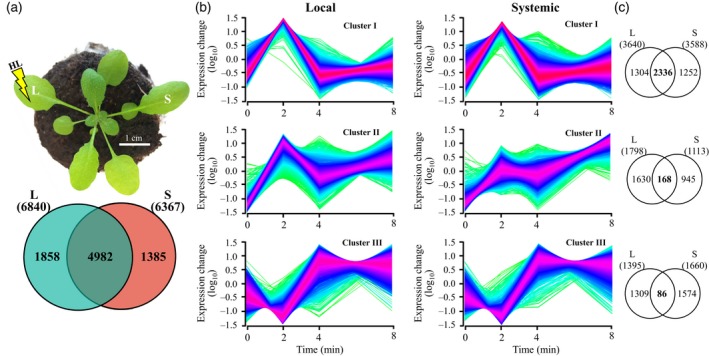
Transcriptomic responses of local (L) and systemic (S) leaves of Arabidopsis plants to local application of excess light stress. (a) The experimental design used (top) and a Venn diagram showing the overlap between local and systemic responses to light stress (bottom). (b) Distinct clusters of transcript expression in local and systemic leaves in response to local application of light stress. (c) Venn diagrams showing the overlap between the different groups of clusters in local and systemic leaves. All Venn diagrams had a hypergeometric testing significance of *P* < 0.001. L, local; S, systemic.

**Figure 2:**
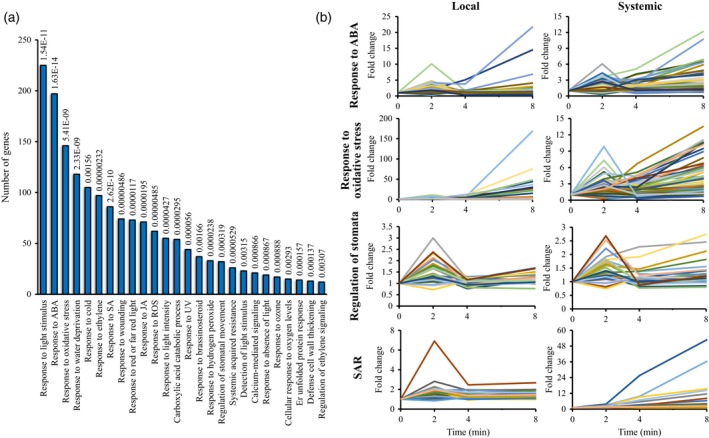
Gene ontology classification and expression pattern of transcripts that accumulate in both local and systemic leaves of Arabidopsis in response to local application of light stress. (a) Gene Ontology annotation of transcripts that accumulate in local and systemic leaves of Arabidopsis in response to light stress. See Table S4 for full description. The *P*‐value for enrichment compared with the genome distribution from Fisher's Exact with FDR multiple test correction is provided for each GO term. (b) Expression pattern of selected gene ontology groups in local and systemic leaves. See Figure S4 for additional classification groups. ABA, abscisic acid; SAR, systemic acquired resistance; SA, salicylic acid; JA, jasmonic acid; ROS, reactive oxygen species.

To study the local and systemic responses of plants to light stress, we subjected a single Arabidopsis leaf to light stress as described in Devireddy *et al*. (2018; local leaf) and sampled it, as well as one systemic leaf, at 0, 2, 4 and 8 min post‐light stress application (Figures 1a and S1). The application of light stress to a single Arabidopsis leaf was previously shown to trigger the ROS wave (Choudhury *et al*., 2018; Devireddy *et al*., 2018; Figure S2). The steady‐state level of 6840 transcripts was significantly enhanced in local leaves in response to light stress, and the steady‐state level of 6367 transcripts was significantly enhanced in systemic leaves in response to the light stress treatment applied to the local leaf (Tables S1, S2; Figure S3). An overlap of 4982 transcripts was found between the transcriptomics response of local and systemic leaves to light stress demonstrating a significant systemic response to this abiotic stress in Arabidopsis (Figure 1a, Table S3). A high representation of transcripts encoding light, ABA, ROS, drought, cold, wounding, heat and other abiotic stress‐response transcripts, including transcripts involved in calcium and systemic acquired response (SAR) signaling was found in this group of transcripts common to local and systemic tissues and demonstrated that the rapid SAA response of Arabidopsis to light stress is complex and includes components involved in the response of plants to many other abiotic conditions (Figure 2a, Table S4).

Cluster analysis of the different transcripts upregulated in response to light stress revealed that a large number of transcripts (over 3500) peaked in their abundance at 2 min and then returned to almost baseline expression levels in local and systemic leaves (Figure 1b). Compared with transcripts that peaked at 2 min, but did not return to baseline level, or transcripts that peaked at 4 min, this group of transcripts also displayed the largest overlap between local and systemic responses to light stress (Figure 1c). This finding demonstrates that a large proportion of the local and systemic responses to light stress in Arabidopsis occurs as early as 2 min following the initiation of stress, highlighting the importance of rapid responses to stress at the local and systemic levels. Many of the hormone‐ and stress‐response transcripts identified in Figure 2a as involved in the response of Arabidopsis to light stress belonged to this group of transcripts that transiently peaked in their expression at 2 min following light stress application (Figures 2b and S1). While the fold change in expression of many of these transcripts (e.g., those associated with ABA and responses to oxidative stress) was lower in systemic leaves compared with local leaves, the fold change in expression of some transcripts (e.g., those associated with SAR to pathogens) was higher in systemic leaves compared with local leaves, and the fold change in expression level of transcripts associated with stomatal function or other plant hormone was similar between local and systemic leaves (Figures 2b and S1).

**Figure 3:**
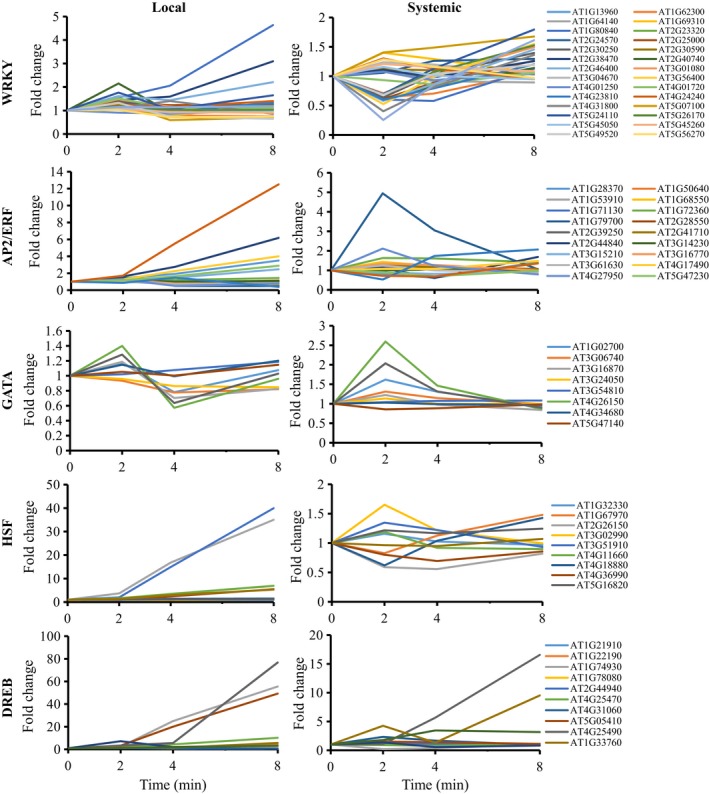
Expression pattern of selected transcription factor families that accumulate in both local and systemic leaves of Arabidopsis in response to local application of light stress. HSF, heat shock factor; DREB, dehydration responsive element binding; AP‐2, activating protein‐2; ERF, ethylene response factor, GATA, (T/A)GATA(A/G)‐binding.

Analysis of the expression pattern of selected transcription factor (TF) families involved in response to different stimuli in local and systemic leaves revealed that their fold change in expression in systemic leaves was lower than that in local leaves (Figures 3 and S5; only transcripts encoding TFs significantly upregulated in both local and systemic tissues are shown). In addition, the kinetics of expression was sometimes different between local and systemic leaves (e.g., heat shock transcription factors; HSFs). In general, many of the different TFs in systemic leaves peaked in their expression (up or down) at 2 min post‐light stress application to local leaves (Figure 3). This pattern could suggest that a systemic signal generated at the local leaf might have reached the systemic leaves and caused a spike in TFs expression within 2 min of light stress application. Potential culprits for such signal may include the ROS/Ca^2+^ wave, electric signals and hydraulic waves that travel at a rate higher than 5 cm min^−1^ (the distance between the local and systemic leaves in our experimental system was approximately 4 cm). The findings presented in Figures 1, 2, 3, S4 and S5 demonstrate that light stress invokes a significant systemic response in Arabidopsis and that this response could be mediated by rapid systemic signaling pathways. Responses to light stress occurring at the local leaves as early as seconds (Suzuki *et al*., 2015) to minutes (Figures 1, 2, 3) could therefore be transmitted within minutes to systemic leaves, highlighting the importance of rapid systemic signaling pathways in mediating SAA in plants.

### Local and systemic responses of *rbohD* plants to excess light stress

**Figure 4:**
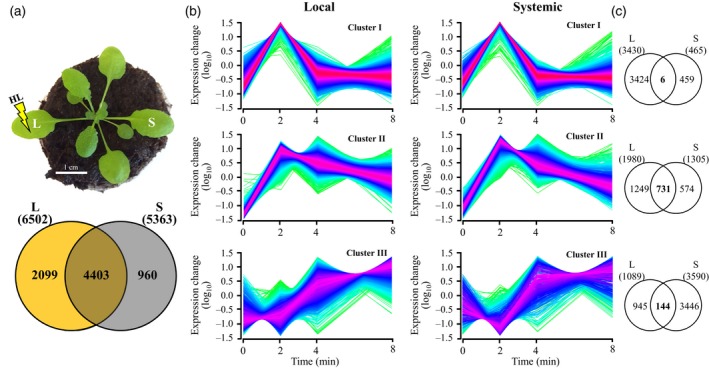
Transcriptomic responses of local (L) and systemic (S) leaves of *rbohD* plants to local application of excess light stress. (a) The experimental design used (top) and a Venn diagram showing the overlap between local and systemic responses to light stress (bottom). (b) Distinct clusters of transcript expression in local and systemic leaves of *rbohD* plants in response to local application of light stress. (c) Venn diagrams showing the overlap between the different groups of clusters in local and systemic leaves. All Venn diagrams had a hypergeometric testing significance of *P* < 0.001. L, local; S, systemic; RBOHD, respiratory burst oxidase homolog D.

To determine what proportion of the systemic response of Arabidopsis to light stress is dependent on the function of the ROS/Ca^2+^ wave, we conducted similar experiments to the ones shown in Figure 1, however, using *rbohD* mutants deficient in the initiation and propagation of the ROS/Ca^2+^ wave (Miller *et al*., 2009). As shown in Figure 4a, 6502 transcripts were significantly upregulated in local leaves of *rbohD* plants in response to light stress, and 5363 transcripts were significantly upregulated in systemic leaves of *rbohD* plants in response to the light stress treatment applied to the local leaf (Tables S5, S6). An overlap of 4403 transcripts was found between the local and systemic leaves of *rbohD* demonstrating that the majority of systemic responses were not diminished in the absence of RBOHD (Figure 4a, Table S7). When the local and systemic‐response transcripts were clustered based on their expression pattern and compared between local and systemic leaves (Figure 4b), a significant effect of RBOHD absence was evident in the expression of rapidly responding transcripts that peak at 2 min and return to almost baseline levels (an overlap of only 6, compared with 2336 in wild type; Figures 1c and 4c, respectively). In addition, the overall number of these rapidly responding transcripts was much lower compared with wild type plants (465 compared with 3588; Figures 1c and 4c, respectively).

**Figure 5:**
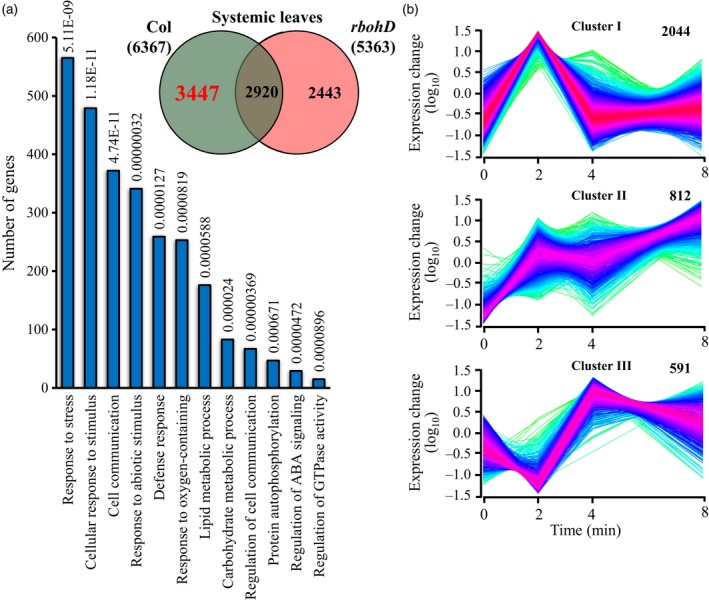
Gene ontology classification and expression pattern of systemic *rbohD*‐dependent transcripts in wild type plants. (a) Gene Ontology annotation of systemic *rbohD*‐dependent transcripts that accumulate in wild type plants in response to local application of light stress. See Table S9 for full description. The *P*‐value for enrichment compared with the genome distribution from Fisher's Exact with FDR multiple test correction is provided for each GO term. (b) Distinct clusters of expression of *rbohD*‐dependent transcripts in systemic leaves of wild type plants in response to local application of light stress. Venn diagram in (a) had a hypergeometric testing significance of *P* < 0.001. ABA, abscisic acid; RBOHD, respiratory burst oxidase homolog D.

A more direct comparison of the systemic response of *rbohD* plants to that of wild type revealed that 3447 transcripts that accumulated in the systemic leaves of wild type plants did not accumulate in the systemic leaves of *rbohD* plants (Figure 5a; Table S8). These transcripts contained a high proportion of transcripts that peaked at 2 min and returned to basal levels (2044), compared with transcripts that peaked at 4 min (591), or gradually increased in their expression from 0 to 8 min (812; Figure 5b). The group of *rbohD*‐dependent 3447 systemic transcripts contained many different transcripts involved in signal transduction, cell‐to‐cell communication and ABA signaling (Figure 5a, Table S9). A high representation of transcripts responding to local treatments of light stress and wounding was further found in all three clusters of the 3447 transcripts, with a high representation of transcripts responding to NaCl and ozone in cluster 2 (Table 1). Interestingly, only cluster 3 contained high representation of transcripts responding to a plant hormone (ABA response; Table 1). Because the ROS/Ca^2+^ wave responds to many different stimuli (Miller *et al*., 2009; Mittler *et al*., 2011), these findings suggest that transcripts that are not related to the ROS/Ca^2+^ wave may also be found in this list of *rbohD*‐dependent systemic transcripts.

**Table 1:** Response of *rbohD*‐dependent (3447) and ROS wave‐associated (82) systemic transcripts to different stresses, hormones and stimuli. Top: Response of *rbohD*‐dependent (3447) and ROS wave‐associated (82) systemic transcripts to different abiotic and biotic stresses. Bottom: Response of *rbohD*‐dependent (3447) and ROS wave‐associated (82) systemic transcripts to different hormones, reactive oxygen species and external ATP

	3447 transcripts			82 transcripts
	Cluster I	Cluster II	Cluster III	
Total	2040	812	591	82
Abiotic stresses				
Drought	153 (7.5%)	39 (4.8%)	89 (15.05%)	6 (7.31%)
Cold	84 (4.11%)	81 (9.97%)	19 (3.21%)	30 (36.58%)
Heat	151 (7.4%)	70 (8.62%)	57 (9.64%)	17 (20.73%)
High light	1474 (72.25%)	638 (78.57%)	333 (56.34%)	62 (75.6%)
NaCl	40 (1.96%)	139 (17.11%)	37 (6.26%)	34 (41.46%)
Ozone	42 (2.05%)	137 (16.87%)	35 (5.92%)	19 (23.17%)
Wounding	290 (14.21%)	330 (40.64%)	136 (23.01%)	49 (59.75%)
Incompatible bacterial pathogen	22 (1.07%)	80 (9.85%)	13 (2.19%)	15 (18.29%)

	3447 transcripts			82 transcripts
	Cluster I	Cluster II	Cluster III	
Total	2040	812	591	82
Hormone/ROS				
ABA	77 (3.77%)	58 (7.14%)	67 (11.33%)	12 (14.63%)
ACC	17 (0.83%)	7 (0.86%)	3 (0.5%)	0 (0%)
Brassinolide	26 (1.27%)	13 (1.6%)	10 (1.69%)	6 (7.31%)
Cytokinin	24 (1.17%)	3 (0.36%)	8 (1.35%)	1 (1.21%)
Gibberellin	7 (0.34%)	4 (0.49%)	0 (0%)	1 (1.21%)
Indole‐3‐acetic acid	50 (2.45%)	23 (2.83%)	14 (2.36%)	8 (9.75%)
Methyl jasmonate	59 (2.89%)	30 (3.69%)	31 (5.24%)	13 (15.85%)
SA	4 (0.19%)	27 (3.32%)	14 (2.36%)	2 (2.43%)
eATP	13 (0.63%)	31 (3.81%)	10 (1.69%)	15 (18.29%)
H_2_O_2_	40 (1.96%)	91 (11.2%)	39 (6.59%)	82 (100%)
O_2_^–^	24 (1.17%)	23 (2.83%)	12 (2.03%)	4 (4.87%)
^1^O_2_	11 (0.53%)	43 (5.29%)	10 (1.69%)	15 (18.29%)
Ca^+2^	64 (3.14%)	60 (7.39%)	35 (5.92%)	14 (17.07%)

ABA, abscisic acid; ACC, 1‐aminocyclopropane‐1‐carboxylic acid; SA, salicylic acid; eATP, external ATP.

### Response of plants to external H_2_O_2_ and overlap with systemic responses to excess light

**Figure 6:**
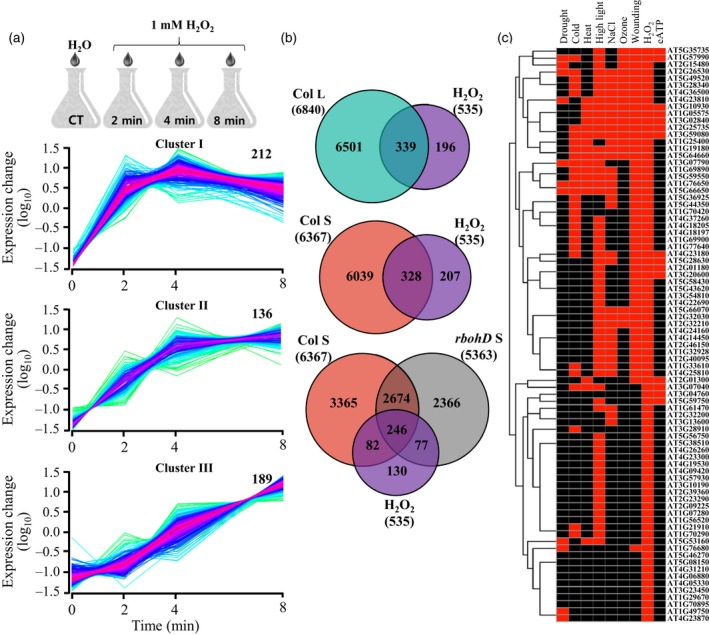
Identification of *rbohD*‐dependent systemic transcripts significantly enhanced in their expression in response to H_2_O_2_. (a) Experimental design of the H_2_O_2_ treatment experiment (top) and distinct clusters of expression of H_2_O_2_–response transcripts. (b) Venn diagrams showing the overlap between transcripts enhanced in their expression in local leaves of wild type plants and seedlings treated with 1 mm H_2_O_2_ (top); Venn diagrams showing the overlap between transcripts enhanced in their expression in systemic leaves of wild type plants and seedlings treated with 1 mm H_2_O_2_ (middle); and Venn diagrams showing the overlap between transcripts significantly accumulated in systemic leaves of wild type plants, systemic leaves of *rbohD* plants and seedlings treated with 1 mm H_2_O_2_ (bottom). (c) Heat map showing the response of the 82 *rbohD*‐dependent and H_2_O_2_‐response systemic transcripts to different stress conditions and signals. All Venn diagrams had a hypergeometric testing significance of *P* < 0.001. L, local; S, systemic; eATP, external ATP.

To identify transcripts more intimately associated with the ROS/Ca^2+^ wave among the 3447 transcripts, we conducted experiments in which we treated Arabidopsis seedlings growing in liquid culture with 1 mm H_2_O_2_. This treatment was chosen to mimic the entry of H_2_O_2_ that accumulates at the apoplast during the propagation of the ROS wave into cells. We used the same time‐course design (0, 2, 4 and 8 min) and conducted RNA‐Seq analysis to identify transcripts enhanced in their expression during this response. As shown in Figure 6a, the steady‐state level of 535 transcripts was enhanced in response to the application of H_2_O_2_, and these transcripts could be divided into three clusters based on their rate of response, with 212 transcripts showing a significant response within 2 min (Figure 6a; Table S10). Of the 535 H_2_O_2_–responsive transcripts, 339 and 328 transcripts were also found to be upregulated in response to light stress in local or systemic leaves, respectively (Figure 6b). Of the 328 transcripts common to H_2_O_2_ and light stress treatment of systemic leaves of wild type plants, 82 transcripts were *rbohD* dependent (Figure 6b; Table S11). These transcripts contained a high proportion of transcripts responsive to many different abiotic stresses (cold, heat, excess light, salinity, ozone, wounding and pathogen infection), ABA, externally applied ATP (eATP), methyl jasmonate, calcium, and singlet oxygen (Figure 6c; Table 1; Suzuki *et al*., 2015; Whalley and Knight, 2013; Chen *et al*., 2017), demonstrating many of the expression features that are expected of true ROS/Ca^2+^ wave‐associated transcripts.

### Functional analysis of ROS wave‐associated transcripts

**Figure 7:**
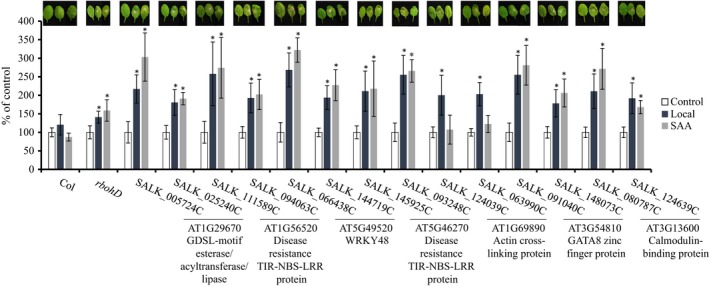
Functional analysis of selected *rbohD*‐dependent systemic transcripts in Arabidopsis. Light stress‐induced cell injury in two independent knockout mutants for seven different genes encoding *rbohD*‐dependent systemic transcripts. Cell injury was measured following application of light stress to a local leaf (local), and following pretreatment of local leaves and the application of light stress to systemic leaves (SAA). Two independent alleles for each gene were subjected to light stress and cell injury was measured by electrolyte leakage. *P < 0.05. AT1G69890, Actin cross‐linking protein; AT3G13600, Calmodulin‐binding family protein; AT3G54810, GATA8 type zinc finger protein; AT1G56520 and AT5G46270, Disease resistance TIR‐NBS‐LRR family protein; AT5G49520, WRKY48 transcription factor; AT1G29670, GDSL‐motif esterase/acyltransferase/lipase. SAA, systemic acquired acclimation.

To determine whether some of the 82 ROS/Ca^2+^ wave‐associated transcripts play a role in the local or systemic response of Arabidopsis to light stress, we obtained and tested two independent confirmed knockout lines for seven of the genes that encode these transcripts (AT1G69890, an actin cross‐linking protein; AT3G13600, a calmodulin‐binding family protein; AT3G54810, a GATA8 protein containing a GATA type zinc finger; AT1G56520 and AT5G46270, two TIR‐NBS‐LRR class disease resistance proteins; AT5G49520, a WRKY48 transcription factor; AT1G29670, a GDSL‐motif esterase/acyltransferase/lipase; mutants were chosen based on availability from TAIR; https://www.arabidopsis.org/; Expression pattern for these selected transcripts are shown in Figures S6 and S7) and subjected them to light stress. As shown in Figure 7, six of these genes were required for light stress acclimation of local or systemic leaves to light stress, whereas one of these genes (AT1G69890, an actin cross‐linking protein) was primarily required for the acclimation of local leaves to light stress. These results demonstrate that some of the genes identified as associated with the ROS/Ca^2+^ wave play an important role in light stress acclimation and are required for the SAA of Arabidopsis to light stress.

### Inhibition of ROS wave‐associated transcripts by DPI

**Figure 8:**
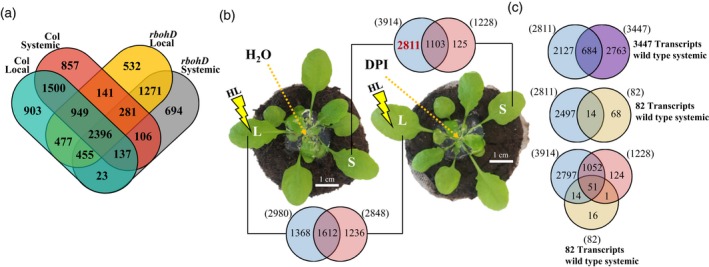
Suppression of light‐induced systemic transcripts by diphenyleneiodonium (DPI). (a) Venn diagram showing the overlap between local and systemic responses to light stress in wild type and *rbohD* plants. (b) Venn diagrams and images showing the position of water or DPI application (in agar) and the overlap between local and systemic responses to light stress in water or DPI‐treated plants. (c) Venn diagrams showing the overlap between the DPI‐suppressed systemic transcripts (2811) and *rbohD*‐dependent systemic transcripts (3447; Top), the DPI‐suppressed systemic transcripts (2811) and the 82 *rbohD*‐dependent and H_2_O_2_‐response systemic transcripts (middle), and the 82 *rbohD*‐dependent systemic transcripts that are also H_2_O_2_‐response transcripts, HL systemic‐response transcripts (3914) and HL systemic‐response transcripts that are not inhibited by DPI (1228; Bottom). Venn diagrams in (b) and (c) had a hypergeometric testing significance of *P* < 0.001. DPI, diphenyleneiodonium; HL, high lights; L, local; S, systemic.

Although *rbohD* plants are deficient in their basal and systemic acclimation to light stress (Figure 7), and display reduced systemic responses to the local application of light stress (Figures 4, 5, 6), they lack expression of the RBOHD protein in both local and systemic tissues. The lack of RBOHD in local tissues could alter some of the local responses to light stress in these plants potentially affecting systemic responses and hampering our attempts to identify ROS/Ca^2+^ wave‐associated transcripts. As shown in Figure 8a, the expression of 532 and 694 transcripts was enhanced in local and systemic tissues of *rbohD* plants in response to light stress, respectively, with an additional 1271 transcripts enhanced in both systemic and local tissues of *rbohD* plants. None of these transcripts was detected in wild type plants (Figure 8a).

To overcome this potential problem and to complement our analysis of wild type and *rbohD* plants (Figures 4, 5, 6), we conducted pharmacological experiments using diphenyleneiodonium (DPI) to block the progression of the ROS wave. DPI was previously shown to block the ROS wave and SAA to light stress, validating this approach (Miller *et al*., 2009; Suzuki *et al*., 2013; Devireddy *et al*., 2018). As shown in Figure 8b, DPI or water was applied to the midpoint between local and systemic tissues and the tissues were sampled for RNA‐Seq analysis at 0 and 8 min post‐light stress application. Compared with the application of water, DPI blocked the expression of 2811 transcripts in systemic tissues (Table S13), and 1368 transcripts in local tissues (Table S12), 8 min following the application of light stress to the local tissue. When compared with the 3447 *rbohD*‐dependent systemic transcripts (Figure 5a), or to the 82 *rbohD*‐dependent and H_2_O_2_‐induced transcripts (Figure 6c), an overlap of 684 and 14 transcripts was found between the DPI‐suppressed transcripts and these two groups respectively (Figure 8c). Interestingly, 51 out of the 82 *rbohD*‐dependent and H_2_O_2_‐enhanced transcripts were not suppressed by the DPI treatment (Figure 8c), demonstrating the potential limits of this approach.

### Putative role for TFs associated with the ROS wave

**Figure 9:**
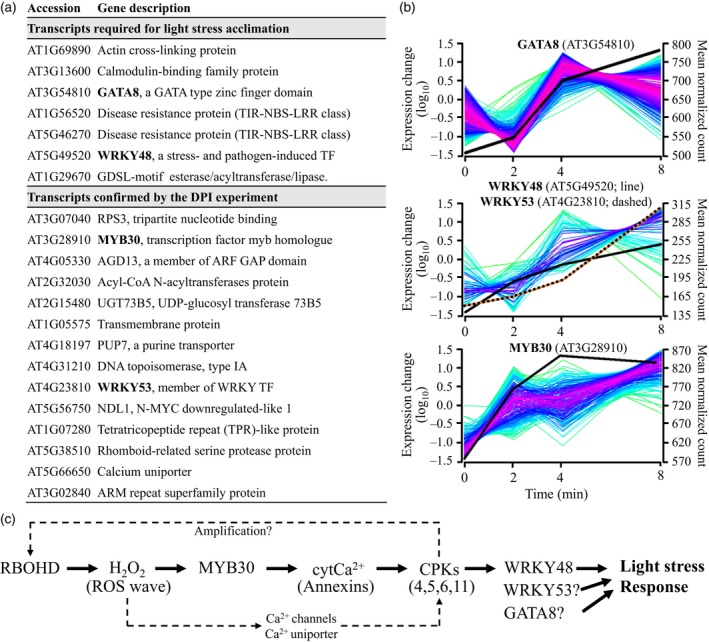
Short list of ROS wave‐associated transcripts, expression pattern of *rbohD*‐dependent systemic transcripts with WRKY, MYB and GATA binding elements in their promoters and a model for the putative function of MYB30. (a) A short list of ROS‐wave‐associated transcripts each confirmed by two independent methods. All transcripts are *rbohD*‐dependent systemic transcripts that are either required for systemic plant acclimation to light stress (top), or suppressed in their expression by DPI (bottom). (b) Distinct clusters of expression of systemic transcripts with WRKY, MYB and GATA binding elements in their promoters. (c) A putative model for the function of MYB30 in mediating or amplifying the ROS wave. See text for more details. CPKs, calcium‐dependent kinases; cytCa^2+^, cytosolic Ca^2+^; BROHD, respiratory burst oxidase homolog D; ROS, reactive oxygen species.

Taking advantage of the different complementary approaches used in our study (i.e., comparing wild type to *rbohD*, pharmacology experiments, and functional analysis of mutants), we complied a short list of putative light stress‐induced ROS/Ca^2+^ wave‐associated transcripts (Figure 9a). This list includes only transcripts that were confirmed by two independent methods (*rbohD*–wild type comparison and functional analysis of mutants, or *rbohD*–wild type comparison and DPI experiments), and includes 21 different transcripts. As shown in Figure 9a, the list includes four different TFs (GATA8, AT3G54810; WRKY48, AT5G49520; WRKY53, AT4G23810; and MYB30, AT3G28910) associated with the ROS/Ca^2+^ wave under these conditions. To test whether some of the target genes of these TFs are also expressed in systemic leaves of plants subjected to light stress, we identified all transcripts encoded by genes that contain putative binding sites for these TFs in the list of *rbohD*‐dependent transcripts significantly upregulated in systemic leaves in response to light stress (Table S8) and clustered them based on their expression pattern. As shown in Figure 9b, many potential target genes for the four different TFs could be found within this list, highlighting the potential role that they could play in regulating systemic responses to light stress.

## Discussion

The context and dynamics of the transcriptomic response of plants to excess light stress was addressed in a number of time‐course studies, with the majority of these using tens of minutes (>30) to hours (≥1) as their first sampling time point (Suzuki *et al*., 2013, 2015; Crisp *et al*., 2017; Kollist *et al*., 2018). We previously showed that transcriptional responses to light stress initiate as early as 20–60 sec post‐light stress application and include hundreds of transcripts, some of these encoded by genes essential for light stress acclimation (Suzuki *et al*., 2015). In addition, we demonstrated that ABA‐dependent physiological responses, such as stomatal movements, occur in plants within the first minute of light stress application in both local and systemic leaves, and that the systemic signal that mediate this rapid systemic stomatal response is dependent on the function of the ROS/Ca^2+^ wave (Devireddy *et al*., 2018). Rapid transcriptomics responses within the minutes range were also recently reported in plants during recovery from light stress (Crisp *et al*., 2017). Here we show that a significant transcriptomics response involving thousands of transcripts is mounted by local and systemic leaves of Arabidopsis plants within minutes of light stress application to a local leaf (Figure 1). The high degree of similarity between the local and systemic transcriptional responses identified by our study (an overlap of 4982 transcripts; Figure 1a), and the rich context of stress‐, acclimation‐ and defense‐associated transcripts within this group of transcripts (Figures 2 and S1) suggest that this rapid response could lead to a successful SAA response. Indeed, wild type plants, but not *rbohD* or mutants impaired in some of the ROS/Ca^2+^ wave‐associated transcripts were able to acclimate successfully to local or systemic light stress following a pretreatment of local leaves by light stress (Figure 7). The identification of such a large number of transcripts upregulated within minutes of light stress application in local and systemic leaves highlights the importance of rapid transcriptional responses in plants, and suggests that rapid local and systemic responses play a key role in the acclimation of plants to light stress.

Many ABA‐response transcripts, as well as transcripts involved in stomatal regulation, are included within the group of transcripts that accumulate in local and systemic leaves within minutes of light stress application (Figures 2 and S1). This finding is in agreement with our previous study that identified light stress‐induced rapid stomatal responses in local and systemic leaves (Devireddy *et al*., 2018). The dependence of the systemic stomatal response on RBOHD and the ROS/Ca^2+^ wave (Devireddy *et al*., 2018) is also in agreement with our findings that expression of many of the rapid and transient response transcripts accumulating in systemic leaves in response to light stress is dependent on the function of the RBOHD protein (Figure 4). Interestingly, compared with the 4982 transcripts common to local and systemic leaves, which contained many different hormone‐response transcripts involved in ABA, ethylene, salicylic acid (SA), and brassinosteroid (BR) responses (Figures 2 and S1), the group of 3447 systemic transcripts we identified as dependent on RBOHD function was primarily enriched in ABA‐response transcripts (Figure 5). These findings further highlight the intimate link between ABA, the ROS/Ca^2+^ wave and responses to light stress (Galvez‐Valdivieso *et al*., 2009; Gorecka *et al*., 2014; Mittler and Blumwald, 2015).

Although many of the transcripts upregulated in systemic leaves in response to light stress applied to a local leaf were also upregulated in local leaves (Figure 1), the kinetics and amplitude of the systemic response to light stress were different than that of the local response (Figures 2, 3 and S4). In general, both local and systemic responses of many TFs, and hormone‐ and stress‐response transcripts peaked in their expression in local and systemic leaves at 2 min following light stress application, however the fold changes in expression in systemic leaves were much lower compared with those in local leaves (Figures 2, 3 and S4). Lower fold amplitude of expression in systemic leaves was observed with many TFs, ABA, ethylene, and oxidative stress‐response transcripts, but not with JA, SA and BR response transcripts, or transcripts involved in stomatal regulation (Figures 2, 3 and S4). In addition, transcripts involved in systemic responses to pathogens (SAR) were primarily upregulated in systemic leaves compared with local leaves. The context and timing of the systemic response to light stress therefore demonstrates high specificity to light stress, which is already evident in systemic leaves as early as 2 min following the application of light to a local leaf. Our finding that many of these early and transient responses to light stress in systemic leaves are suppressed, or delayed in *rbohD* plants (Figure 4) highlights the key role that the ROS/Ca^2+^ wave plays in promoting SAA to light stress in plants. Priming of a systemic leaf to become acclimated to a particular stress during SAA involves, therefore, thousands of transcripts that display a rapid and transient expression pattern driven by the ROS wave. These could have the same abundance as that in local leaves, or lower, involve many hormone‐response and TF‐encoding transcripts (Figures 2 and S4), and lead to successful acclimation to light stress (Figure 7).

The group of ROS/Ca^2+^ wave‐associated transcripts identified by our study includes four transcriptional regulators (GATA8, WRKY48, WRKY57 and MYB30; Figure 9). In addition, it includes transcripts involved in calcium regulation (calmodulin and a calcium uniporter), responses to pathogens (two TIR‐NBS‐LRRs and a UDP‐glucosyl transferase), microtubule organization (NDL1 and an actin cross‐linking protein), and transcripts involved in lipid signaling (GDSL esterase/acyltransferase/lipase), all H_2_O_2_ response transcripts (Figure 9a). GATA8 is a zinc finger TF found to be a positive regulator of Arabidopsis seed germination (Liu *et al*., 2005), and WRKY48 and WRKY57 were previously shown to regulate responses to pathogen infection and drought (Xing *et al*., 2008; Van Eck *et al*., 2014; Sun and Yu, 2015). By contrast, MYB30 was identified as a central regulator of calcium and ROS responses in Arabidopsis (Liao *et al*., 2017). It was shown to be a key regulator of an H_2_O_2_‐response gene network that leads to inhibition of root cell elongation during oxidative stress (Mabuchi *et al*., 2018), an important regulator of calcium signaling in response to heat stress (Liao *et al*., 2017), and a key regulator of ABA signaling (Zheng *et al*., 2012). In addition, it was shown to act as a positive regulator of cell death during the hypersensitive response of plants to pathogen attack (Vailleau *et al*., 2002), and to be dependent on SA for its function in pathogen responses (Raffaele *et al*., 2006). Of particular interest for the regulation of the ROS/Ca^2+^ wave is the role of MYB30 in regulating cytosolic calcium (cytCa^2+^) levels in plants. MYB30 was shown to alter cytCa^2+^ in response to H_2_O_2_ by altering the expression of annexins (Liao *et al*., 2017). During the propagation of the ROS/Ca^2+^ wave, MYB30 could therefore respond to elevated levels of cytosolic H_2_O_2_ that result from H_2_O_2_ entering the cell from the apoplast (Miller *et al*., 2009; Mittler *et al*., 2011), alter annexin expression and regulate cytCa^2+^ levels (Figure 9c). This process would, in turn, activate calcium‐dependent kinases (CPKs) that would trigger RBOHD function (Drerup *et al*., 2013; Dubiella *et al*., 2013; Gilroy *et al*., 2014, 2016) as well as the expression of downstream TFs such as WRKYs (Gao *et al*., 2013). This process could act as a positive amplification loop to enhance the ROS signature and trigger or suppress the expression of many different target genes. Because the ROS/Ca^2+^ was shown to depend on the function of calcium channels (Evans *et al*., 2016) and to be very rapid (Miller *et al*., 2009), the function of MYB30 in regulating calcium via regulating gene expression (Liao *et al*., 2017) could represent a potentially later stage in the activation or amplification of the ROS/Ca^2+^ wave. Further studies are required to address the role of MYB30 in mediating, amplifying or maintaining the ROS/Ca^2+^ wave in plants, as well as to address the different downstream target genes that are activated by MYB30 during this process.

Our study reveals that the priming process of a systemic leaf to become acclimated to a potential stress event involves a rapid systemic transcriptomic response that is extensive and includes an early (2 min) and transient stage of transcripts expression. This early stage of expression is dependent on RBOHD and the function of the ROS/Ca^2+^ wave that originates in the stressed local leaf. Our study further reveals that a core set of transcripts is associated with the ROS/Ca^2+^ wave and suggests that some of these transcripts could be involved in linking ROS with calcium signaling and initiate or amplify the ROS/Ca^2+^ wave.

## Experimental procedures

### Plant material and growth conditions

For RNA‐seq analysis of the SAA to light stress, *Arabidopsis thaliana* Col‐0 (cv. Columbia‐0) and *rbohD* knockout plants (Torres *et al*., 2002; Miller *et al*., 2009) were grown in peat pellets (Jiffy‐7, Jiffy, http://www.jiffygroup.com/) at 23°C under short day growth conditions (8‐h light/16‐h dark, 50 μmol m^−2^ s^−1^). For electrolyte leakage (cell injury) assay, selected knockout lines for the 82 genes encoding *rbohD*‐dependent ROS‐responsive transcripts (Table S11) were obtained from ABRC (http://abrc.osu.edu/) and grown together with wild type and *rbohD* knockout seeds under constant light (50 μmol m^−2^ s^−1^). For the RNA‐seq of response to external H_2_O_2_, Col‐0 seedlings were grown in 100 ml of sterile 0.5× MS medium on a shaker under constant light (50 μmol m^−2^ s^−1^) for 5 days.

### Light stress and H_2_O_2_ treatments

Local leaves of 4‐ to 5‐week‐old plants grown under short day growth conditions as described above were exposed to a light intensity of 2000 μmol m^−2^ s^−1^ for periods of 0, 2, 4 or 8 min using a gooseneck light source (ACE I; Schott) as described in Suzuki *et al*. (2013) and Devireddy *et al*. (2018). Local, as well as non‐treated distant (systemic) leaves (Figure S1) were immediately frozen in liquid nitrogen at each of the time points and used for RNA‐seq analysis. Local and systemic leaves from 45–50 different plants (each a technical repeat) were pooled for each time point and the experiment was repeated in three different biological replicates. All experiments were conducted at the same time of day (9–10 am). All plants used for the experiments were of the same age and developmental stage (Figure S1). Four plants were treated and harvested in each batch simultaneously. H_2_O_2_ treatment was conducted by adding 1 mm H_2_O_2_ to 5‐day‐old Col seedlings growing in 0.5× MS medium. Distilled water was added to control seedlings. Seedlings were immediately frozen in liquid nitrogen after 2, 4 or 8 min of H_2_O_2_ treatment. About 100–150 seedlings were used for each time point and the experiment was repeated three times.

### Electrolyte leakage assay

To test the basal tolerance of plants to light stress, an electrolyte leakage assay was performed as described in Suzuki *et al*. (2015) and Devireddy *et al*. (2018) (Figure S8) with some modifications. A fully expanded local leaf of 21‐ to 25‐day‐old plants was exposed to a light intensity of 2000 μmol m^−2^ s^−1^ for 45 min using a gooseneck light source, photographed and sampled for electrolyte leakage measurements as described in Suzuki *et al*. (2015). For measuring SAA to light stress, a single leaf was pretreated for 10 min with a light intensity of 2000 μmol m^−2^ s^−1^. Plants were then incubated for 50 min under controlled conditions. After the recovery period, a systemic leaf was exposed to a light intensity of 2000 μmol m^−2^ s^−1^ for 45 min. Leaves were then photographed and analyzed for electrolyte leakage as described in Suzuki *et al*. (2015). Briefly, leaves were immersed in 10 ml of distilled water in 50‐ml falcon tubes. Samples were shaken at room temperature for 1 h and the conductivity of the water was measured using a conductivity meter. Leaves were then heated to 95°C using a water bath for 20 min, shaken at room temperature for 1 h and the conductivity of the water was measured again. The electrolyte leakage was calculated as the percentage of the conductivity before heating over that after heating.

### Inhibitor studies

To inhibit the propagation of the ROS wave from the local to the systemic leaf, a drop of 0.3% agarose‐containing water or 50 mm diphenyleneiodonium (DPI) was placed at the midpoint between the local and systemic leaves of 4‐ to 5‐week‐old Col‐0 plants for 15 min as described in Devireddy *et al*. (2018). Local tissue was then subjected to light stress for 8 min as described above and both local and systemic leaves were immediately frozen in liquid nitrogen for RNA‐seq. Here, 45−50 different plants were used for each biological replicate, with the experiment repeated three times.

### RNA sequencing and differential gene expression analysis

Total RNA was isolated using TRIzol (Invitrogen Life Technologies, https://www.thermofisher.com/us/en/home/brands/invitrogen.html) according to the manufacturer's instructions and purified using a NucleoSpin RNA Clean‐up kit (Macherey‐Nagel, https://www.mn-net.com/). Initial RNA sample quality was assessed with a Bioanalyzer RNA 6000 Nano Kit (Agilent) using the 2100 Bioanalyzer System (Agilent, https://www.agilent.com/). RNA quantification was performed with a Qubit RNA Broad Range Assay Kit (Invitrogen) using the Qubit 3.0 Fluorometer (Invitrogen, https://www.thermofisher.com/us/en/home/brands/invitrogen.html). RNA libraries were prepared from 1 μg of total RNA and dual‐indexed with a TruSeq Stranded mRNA HT Library Prep Kit (Illumina, https://www.illumina.com/). Resulting cDNA libraries were quantified with a Qubit dsDNA High Sensitivity Assay Kit (Invitrogen) on a Qubit 3.0 Fluorometer (Invitrogen). Fragment length was validated on the 4200 TapeStation System (Agilent) with a TapeStation D1000 Assay Kit (Agilent) prior to library pooling and normalization to a loading concentration of 1.6 pM. Sequencing was performed with four NextSeq High Output 1 × 75 Reagent Cartridges (Illumina) on a NextSeq 500 Sequencing Platform (Illumina) and produced 1.81G (PF) reads with a *Q* score ≥ 93.84%. RNA library construction and sequencing were performed by the BioDiscovery Institute Genomics Center at the University of North Texas, Denton, Texas, USA (http://untgenomicscenter.squarespace.com/).

Single‐end sequenced reads obtained from the Illumina NextSeq500 platform were quality‐tested using FastQC v0.11.7 (Andrews, 2010) and aligned to the reference genome of Arabidopsis (genome build 10) obtained from TAIR (https://www.arabidopsis.org/) using STAR aligner v2.4.0.1 (Dobin *et al*., 2013). Default mapping parameters (10 mismatches/read; nine multi‐mapping locations/read) were used. The genome index was generated using the gene annotation file (gff file) obtained from TAIR (https://www.arabidopsis.org/) for the genome build 10. Raw and processed RNA‐Seq data files were deposited in GEO (https://www.ncbi.nlm.nih.gov/geo/) under the following accession numbers GSE117300, GSE117296, GSE117297, and, GSE117298.

Differential gene expression analysis was carried out using DESeq2 v1.20.0, an R based package available from Bioconductor (Love *et al*., 2014). Transcripts expressing differentially in two (or more) conditions were identified by examining the difference in their abundance under the conditions. The abundance of a transcript is measured as mean normalized count of reads mapping onto the transcript (Love *et al*., 2014). The difference in expression was quantified in terms of the logarithm of the ratio of mean normalized counts between two conditions (log fold change). Differentially expressed transcripts for our experiments were defined as those that have a fold change with an adjusted *P*‐value < 0.05 (negative binomial Wald test followed by a Benjamini−Hochberg correction; both integral to the DESeq2 package). Differentially expressed genes were classified into upregulated or downregulated based on significant positive or negative log fold change values, respectively. Venn diagram overlap was subjected to hypergeometric testing using phyper (R package; Table S15). Smear (Bland–Altman) plots generated in edgeR (R package), and heat maps were generated using the ComplexHeatmap package v1.18.1 available in BioConductor 3.7. Summary statistics reading for the sequencing performed in included in Table S14. Perl scripts used in this study were uploaded in: https://github.com/sohamsg90/RNA-Seq-perl-scripts.

### Clustering

To identify clusters of transcripts with similar expression patterns, we used the Mfuzz v2.40.0 package of R Bioconductor to perform *k*‐means soft clustering (Kumar and Futschik, 2007).

### Gene enrichment analysis

Functional annotations and overrepresentation of GO terms in gene lists of Tables S4 and S9 were performed using PANTHER v9.0 (http://www.pantherdb.org/). PANTHER Overrepresentation tests were performed using GO molecular function, biological process, and, cellular component annotation data sets. Fisher's Exact with FDR multiple test correction was used to compare enrichment to the genome distribution.

### Stress comparison

The overlap between RBOHD‐dependent transcripts enhanced in systemic leaves of Col plants in response to a local light stress treatment and transcripts enhanced in response to a hormone/ROS, namely ABA, ethylene (ACC), brassinolides, cytokinin, gibberellin, auxin (IAA), methyl jasmonate (MJ), salicylic acid (SA), H_2_O_2_, O_2_^−^ or ^1^O_2_ (Davletova *et al*., 2005; Gadjev *et al*., 2006; Nemhauser *et al*., 2006; Scarpeci *et al*., 2008; Blanco *et al*., 2009) and their distribution in the three clusters was determined using an in‐house written Perl script (Table 1). Similarly, the overlap and distribution were also obtained for transcripts that were also enhanced in response to an external H_2_O_2_ treatment. In addition, the overlap of these classes of light‐stress transcripts with the transcripts previously reported to be enhanced in response to other abiotic stresses (Tosti *et al*., 2006; Truman *et al*., 2006; Kleine *et al*., 2007; Huang *et al*., 2008; Larkindale and Vierling, 2008; Matsui *et al*., 2008; Consales *et al*., 2012; Choi *et al*., 2014; Ding *et al*., 2014; Ikeuchi *et al*., 2017) and their distribution in the three clusters was also obtained. Expression heat maps were generated using pheatmap R package (Kolde and Kolde, 2018).

### Promoter analysis

Promoter sequences (1000 bp upstream of gene start) for RBOHD‐dependent genes differentially regulated in systemic leaves of Col plants in response to a local light stress treatment (Table S8) were downloaded from TAIR. Transcription factor binding sites for GATA8 (AT3G54810), WRKY48 (AT5G49520), WRKY53 (AT4G23810), and MYB30 (AT3G28910) were obtained from the Arabidopsis Gene Regulatory Information Server (AGRIS; http://agris-knowledgebase.org/). The occurrence of DNA‐binding elements of the above‐mentioned transcription factors (TFs) in the different promoters was determined using an in‐house Perl script.

## Conflict of Interest

The authors declare no conflict of interest.

## Supporting information


**Figure S1.** Definition of local and systemic leaves used for this study.
**Figure S2.** Response of *Zat12::Luciferase* reporter plants to local application of light stress. Results are presented for three individual plants.
**Figure S3.** Smear (Bland–Altman) plots generated in edgeR for local and systemic leaves at 0 and 2 min and for the H_2_O_2_ treatment at 0 and 8 min.
**Figure S4.** Expression pattern of selected gene ontology groups in local and systemic leaves. See Figure 2.
**Figure S5.** Heatmap representation of Figure 3.
**Figure S6.** Expression pattern (in counts) of the seven transcripts selected for analysis using knockout mutants (Figure 7).
**Figure S7.** Expression pattern (in fold change) of the seven transcripts selected for analysis using knockout mutants (Figure 7).
**Figure S8.** Position of leaves used for electrolyte leakage assay.

**Table S1.** Transcripts significantly elevated in their expression in local leaves in response to light stress.
**Table S2.** Transcripts significantly elevated in their expression in systemic leaves in response to light stress treatment applied to a local leaf.
**Table S3.** List of overlapping transcripts between the transcriptomics response of local and systemic leaves to light stress applied to a local leaf.
**Table S4.** Gene ontology of overlapping transcripts between the transcriptomics response of local and systemic leaves to light stress.
**Table S5.** Transcripts significantly elevated in their expression in local leaves of *rbohD* plants in response to light stress.
**Table S6.** Transcripts significantly elevated in their expression in systemic leaves of *rbohD* plants in response to the light stress treatment applied to a local leaf.
**Table S7.** List of overlapping transcripts between the transcriptomics response of local and systemic leaves of *rbohD* plants to light stress applied to a local leaf.
**Table S8.** Transcripts significantly elevated in their expression in systemic leaves of wild type plants, but not in systemic leaves of *rbohD* plants.
**Table S9.** Gene ontology of transcripts significantly elevated in their expression in systemic leaves of wild type plants, but not in systemic leaves of *rbohD* plants.
**Table S10.** Transcripts significantly elevated in their expression in seedlings in response to the external application of H_2_O_2_.
**Table S11.** List of *rbohD*‐dependent systemic transcripts that are also significantly elevated in their expression in response to the external application of H_2_O_2_.
**Table S12.** List of transcripts suppressed in their local expression by DPI.
**Table S13.** List of transcripts suppressed in their systemic expression by DPI.
**Table S14.** Summary statistics reading for the sequencing performed.
**Table S15.** Summary hypergeometric testing for the different Venn diagrams.
