# Supplementary material for: Identification and characterization of a core set of ROS wave‐associated transcripts involved in the systemic acquired acclimation response of Arabidopsis to excess light
Source: Plant J. 2019 Jan 30;98(1):126–41. doi: 10.1111/tpj.14205 (PMC6850305; doi:10.1111/tpj.14205)
Supplement: Supplementary file 1 — Figure S1. Definition of local and systemic leaves used for this study. Figure S2. Response of Zat12::Luciferase reporter plants to local application of light stress. Results are presented for three individual plants. Figure S3. Smear (Bland–Altman) plots generated in edgeR for local and systemic leaves at 0 and 2 min and for the H2O2 treatment at 0 and 8 min. Figure S4. Expression pattern of selected gene ontology groups in local and systemic leaves. See Figure 2. Figure S5. Heatmap representation of Figure 3. Figure S6. Expression pattern (in counts) of the seven transcripts selected for analysis using knockout mutants (Figure 7). Figure S7. Expression pattern (in fold change) of the seven transcripts selected for analysis using knockout mutants (Figure 7). Figure S8. Position of leaves used for electrolyte leakage assay. [file TPJ-98-126-s001.pdf]

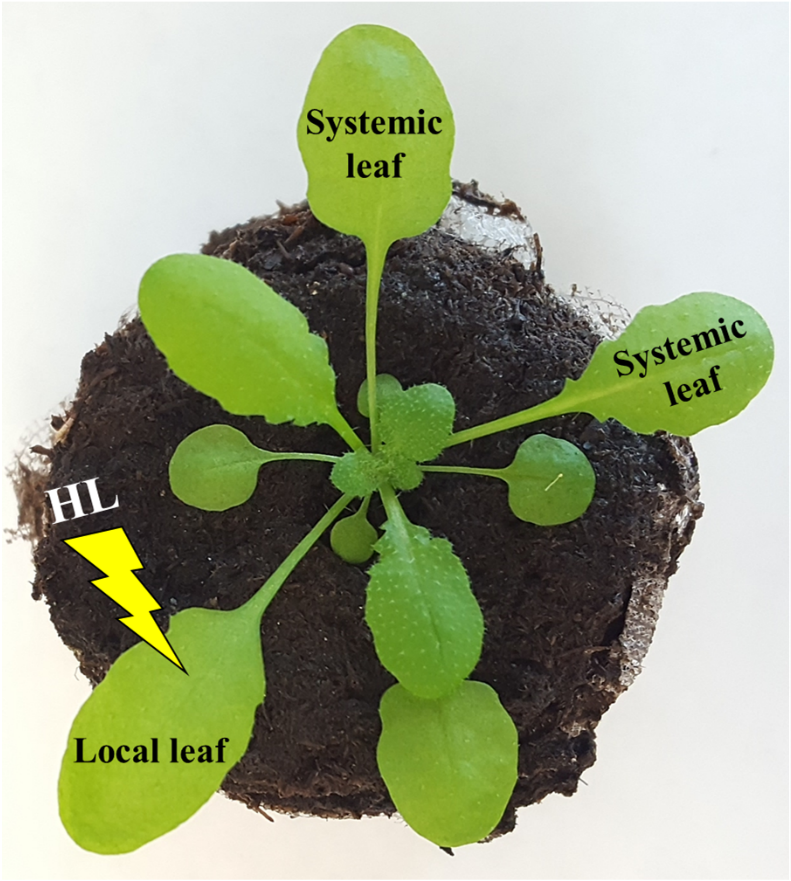

Fig. S1 Definition of local and systemic leaves used for this study.

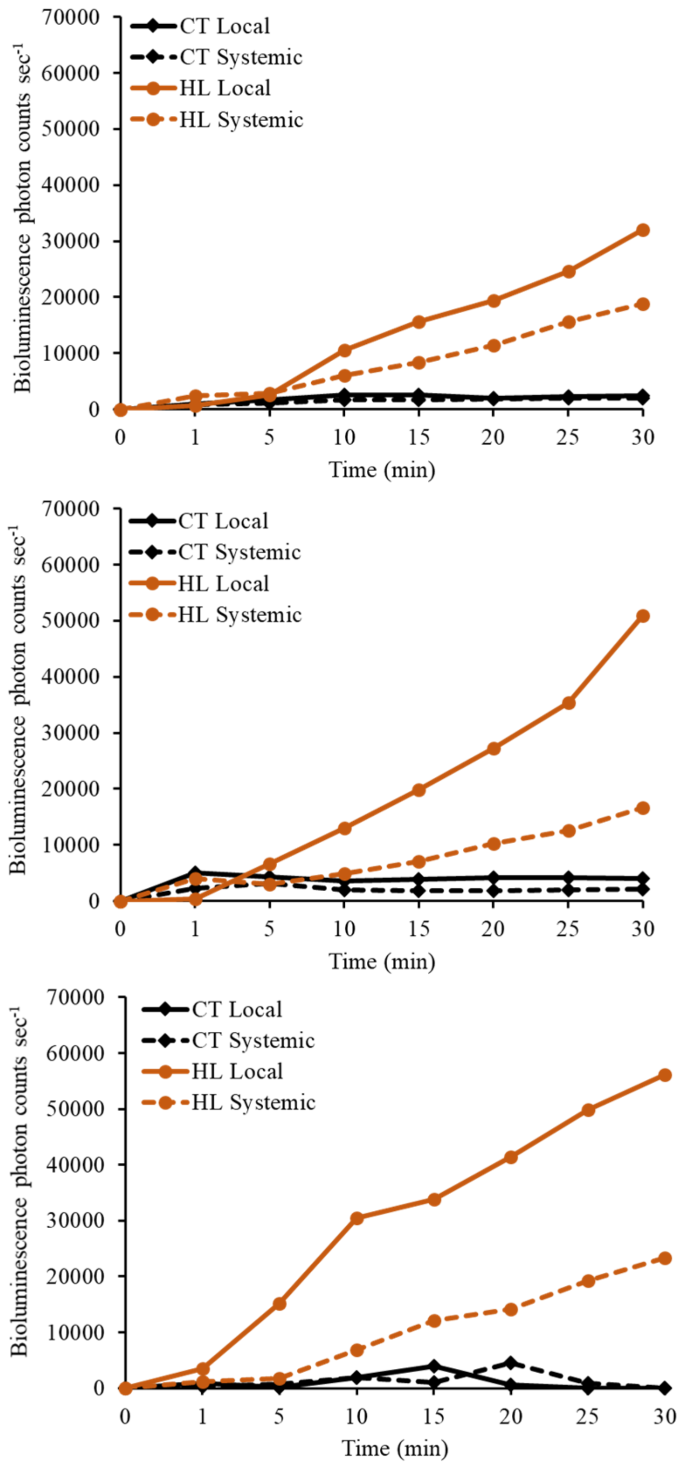

Fig. S2 Response of Zat12::Luciferase reporter plants to local application of light stress. Results are presented for 3 individual plants. Col plants expressing the Zat12::Luciferase reporter construct were grown and imaged with a NightOWL LB983 NC100 (Berthold, https://www.berthold.com/) imager as described in Devireddy et al., 2018.

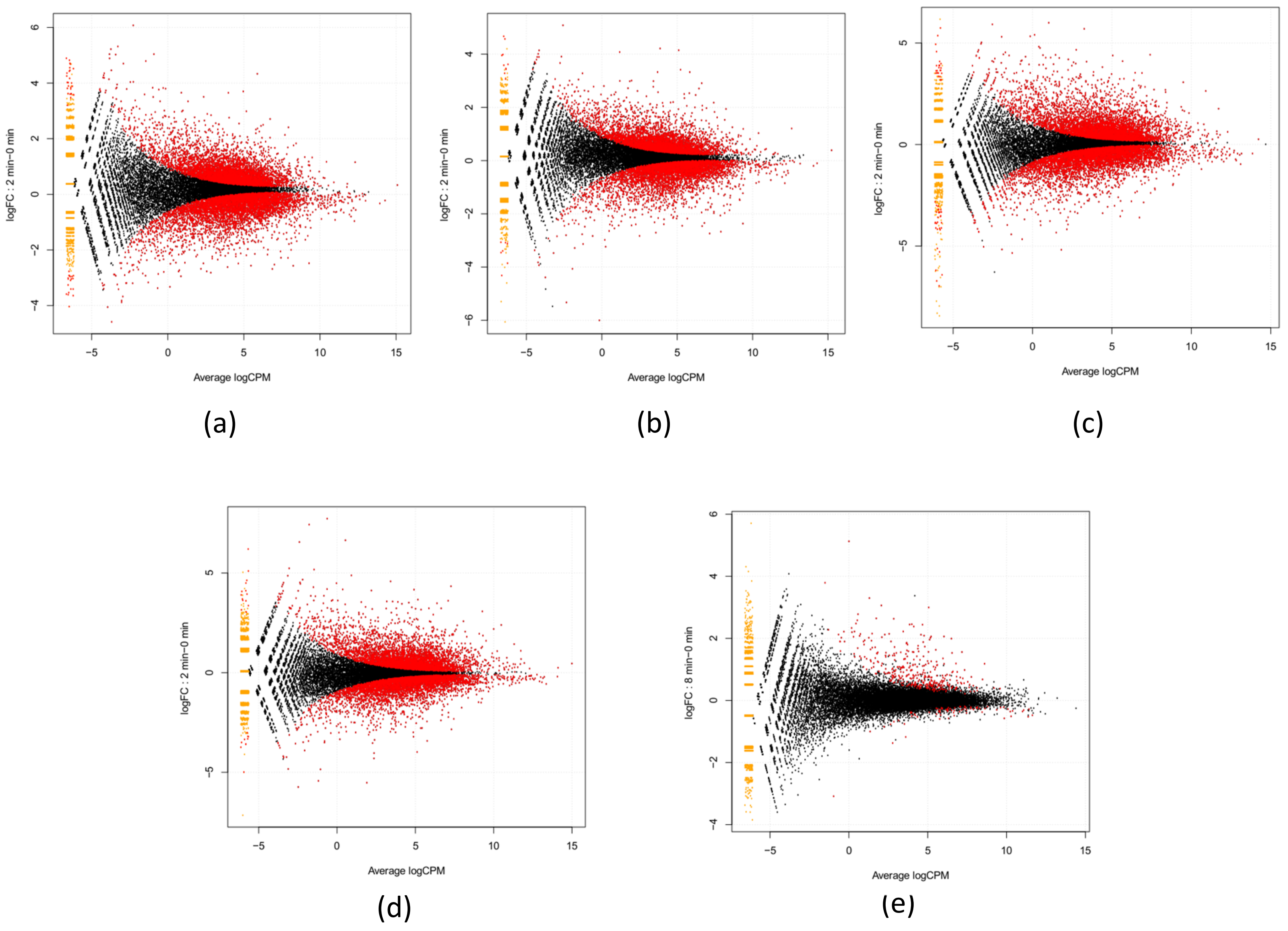

Fig. S3 Smear (Bland–Altman) plots generated in edgeR for local and systemic leaves at 0 and 2 min, and for the H_2O_2 treatment at 0 and 8 min. (a) Col Local Leaf (b) Col systemic leaf (c) rbohD local leaf, (d) rbohD systemic leaf, and (e) Seedlings exposed to H_2O_2.

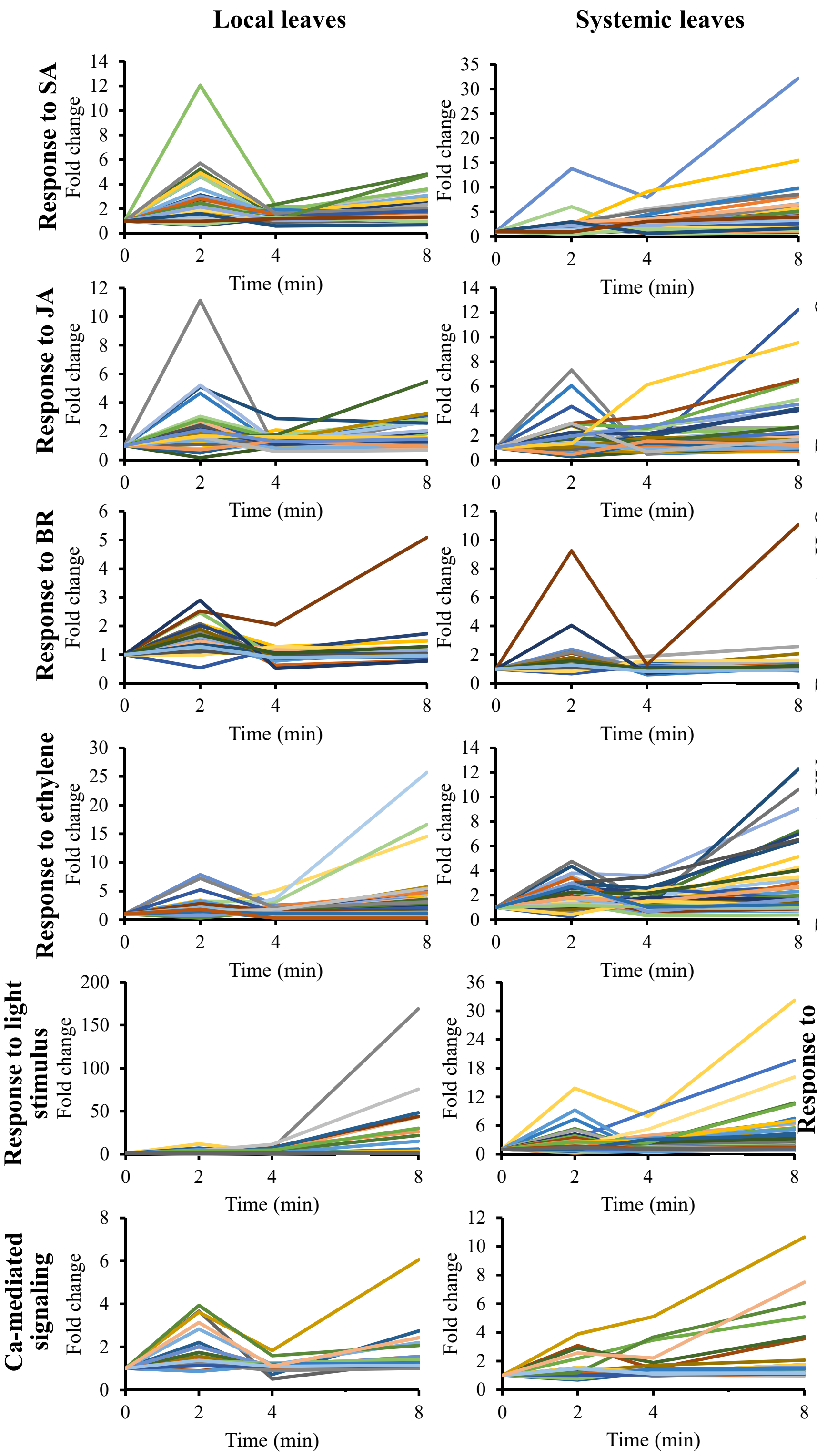

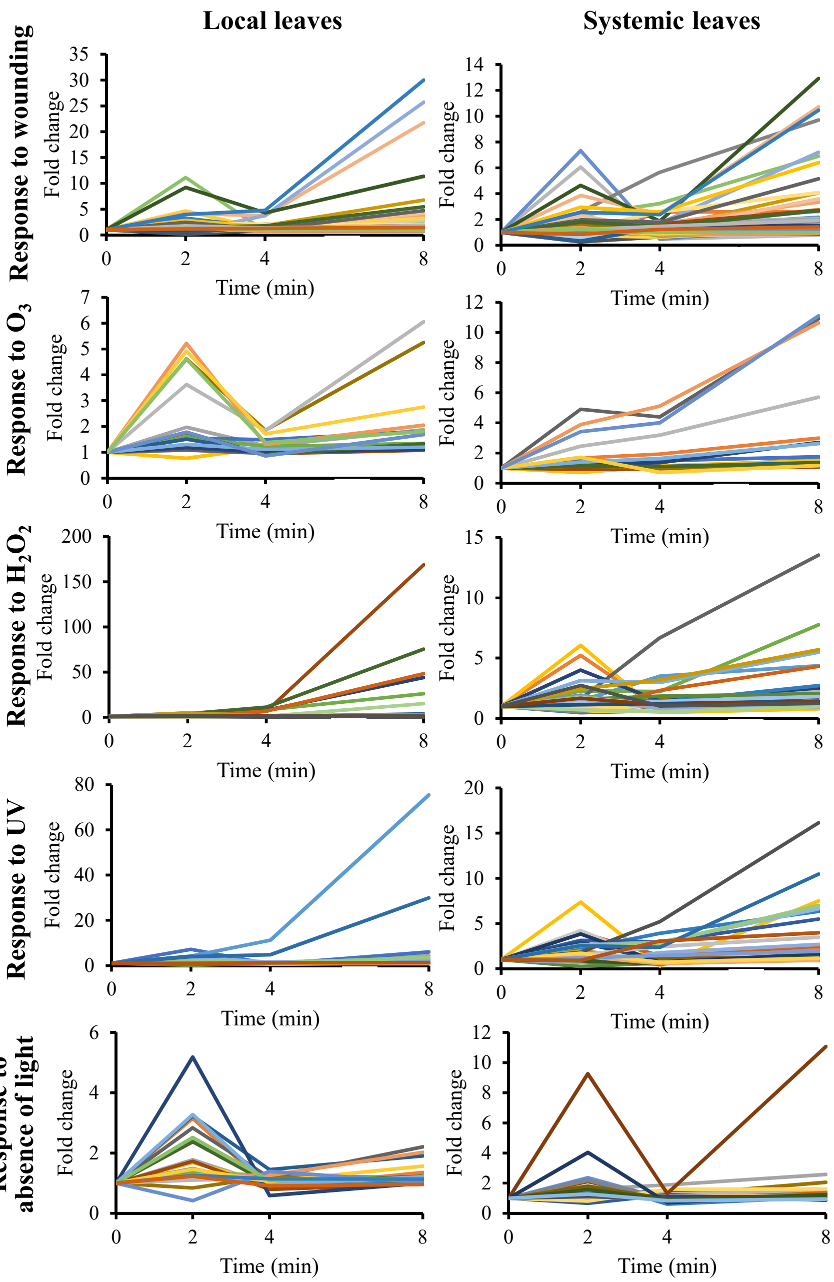

Fig. S4 Expression pattern of selected gene ontology groups in local and systemic leaves. See Fig. 2.

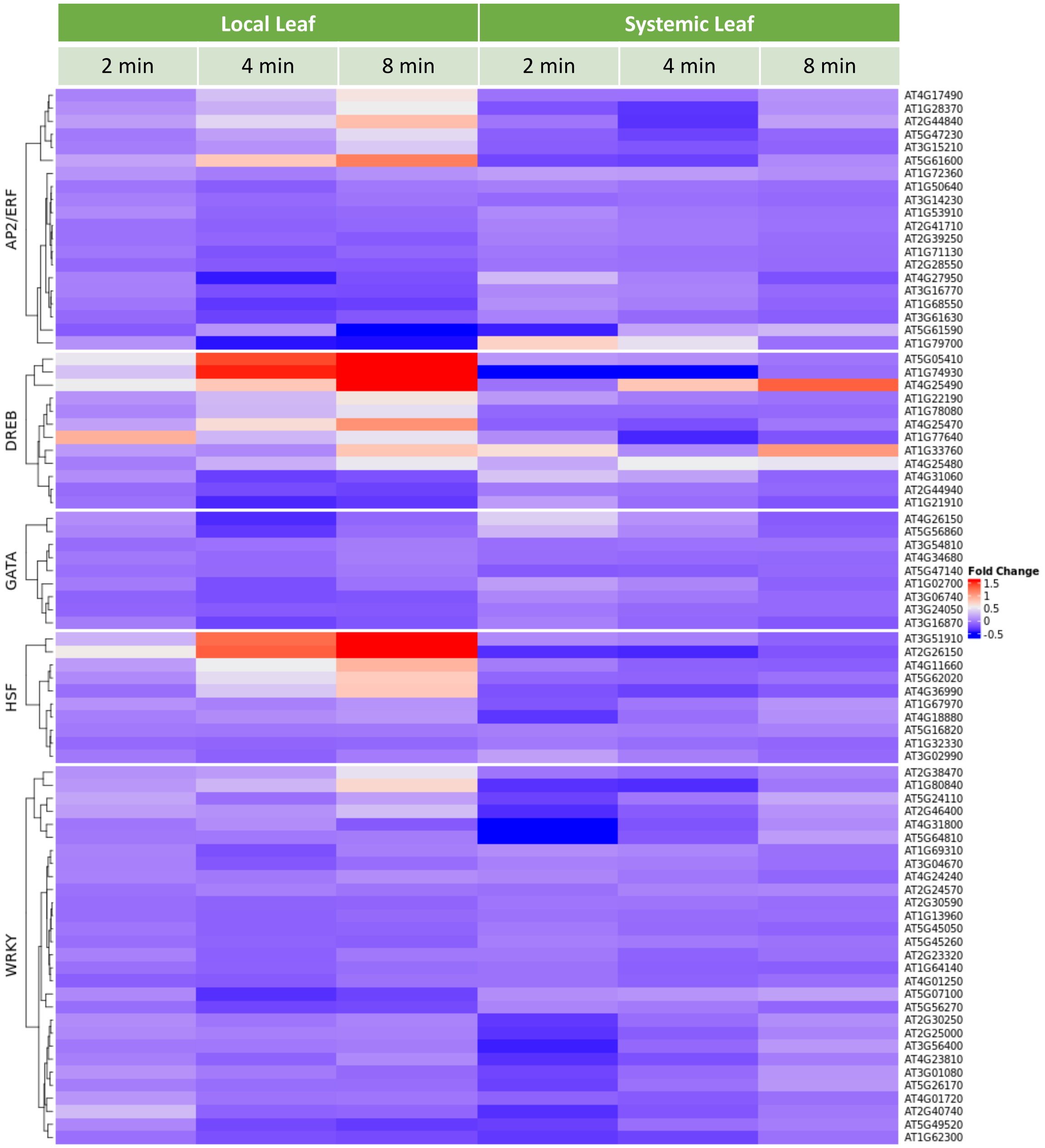

Fig. S5 Heatmap representation of Figure 3. Fold-changes were log2 transformed, and used to generate the figure using the ComplexHeatmap package version 1.18.1 available in BioConductor 3.7. Rows were initially grouped by their transcription factor family, and then separately subjected to hierarchical clustering with complete linkage cluster analyses of their Euclidean distance.

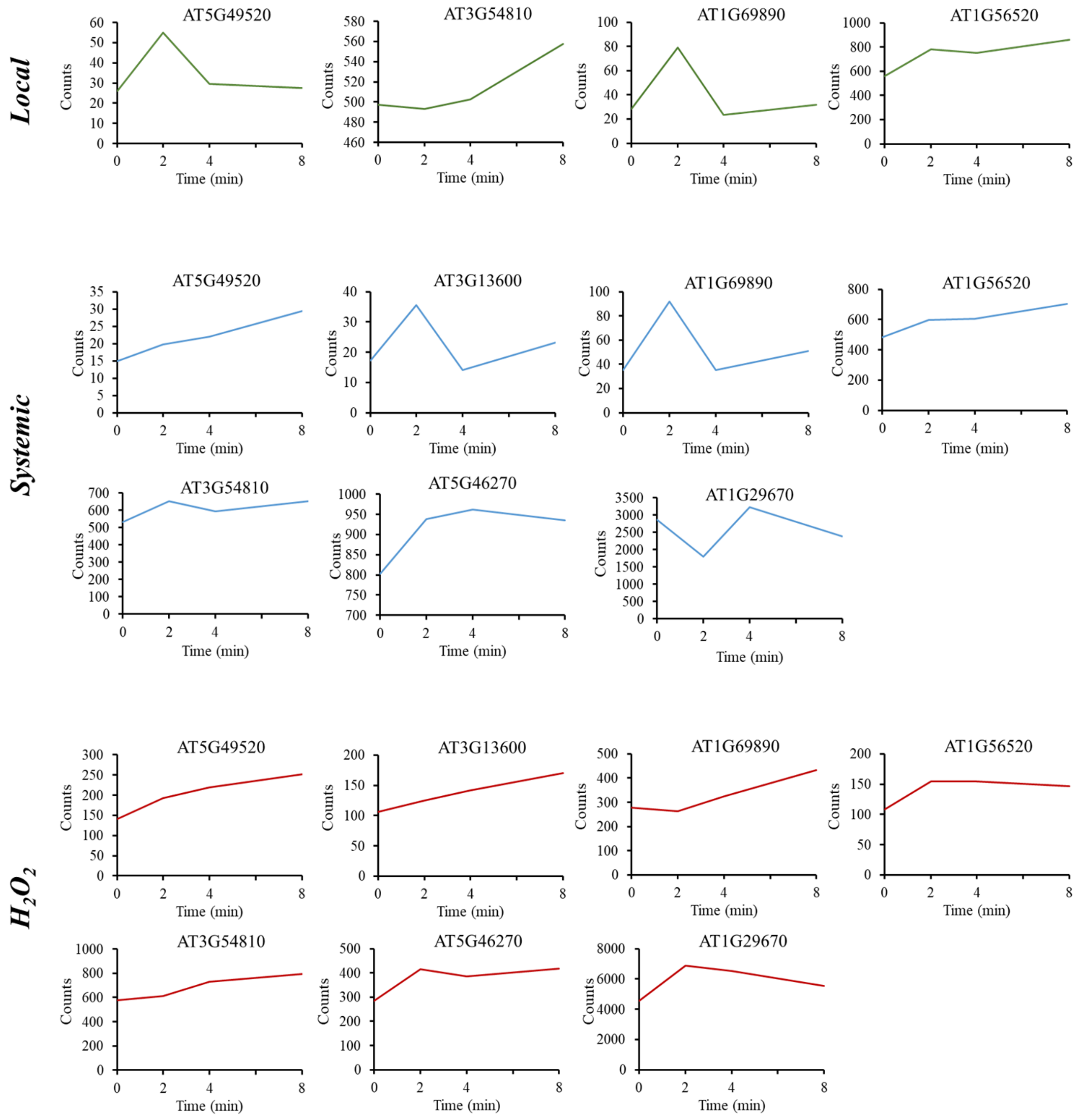

Fig. S6 Expression pattern (in counts) of the 7 transcripts selected for analysis using knockout mutants (Figure 7).

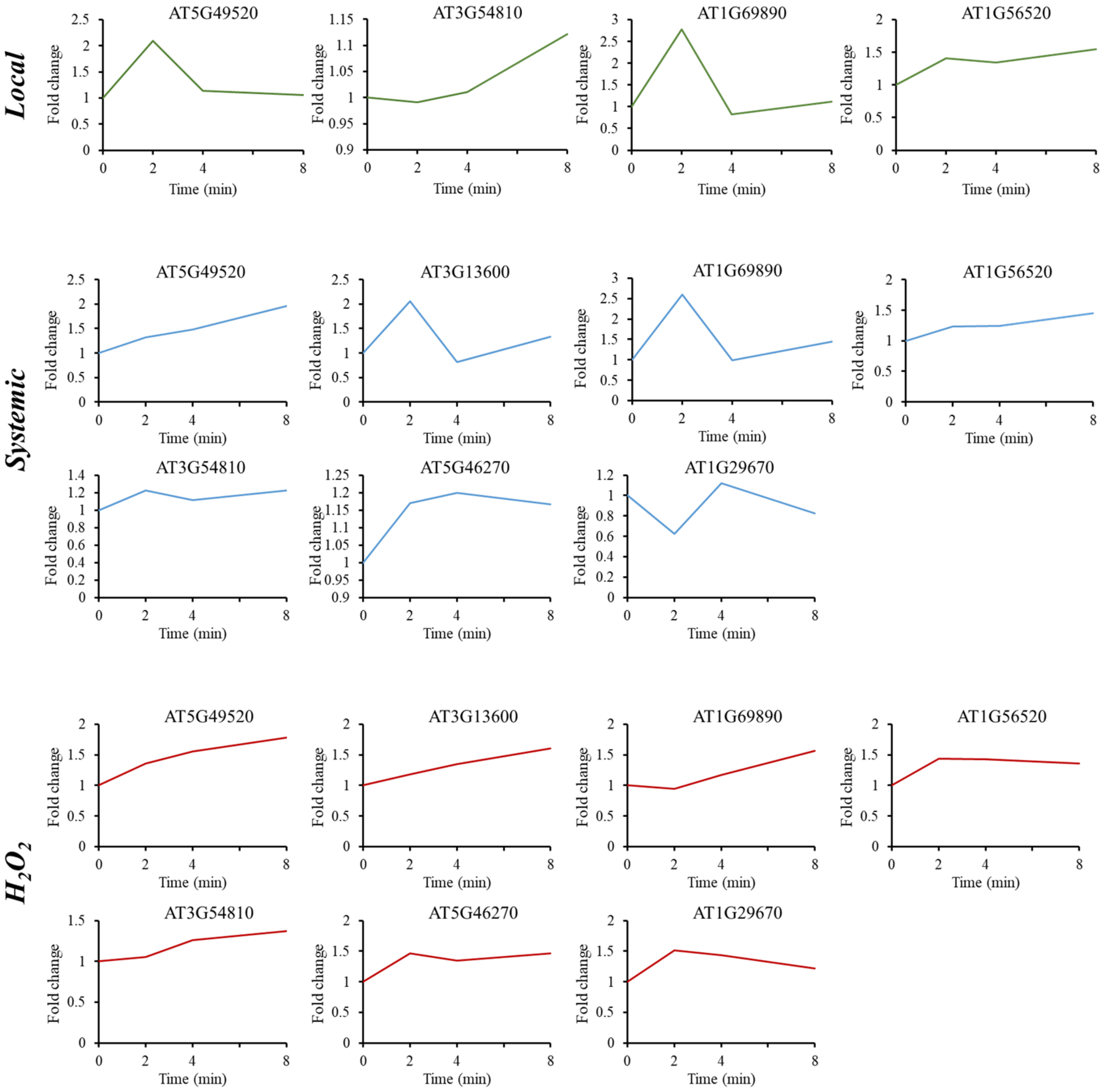

Fig. S7 Expression pattern (in fold) of the 7 transcripts selected for analysis using knockout mutants (Figure 7).

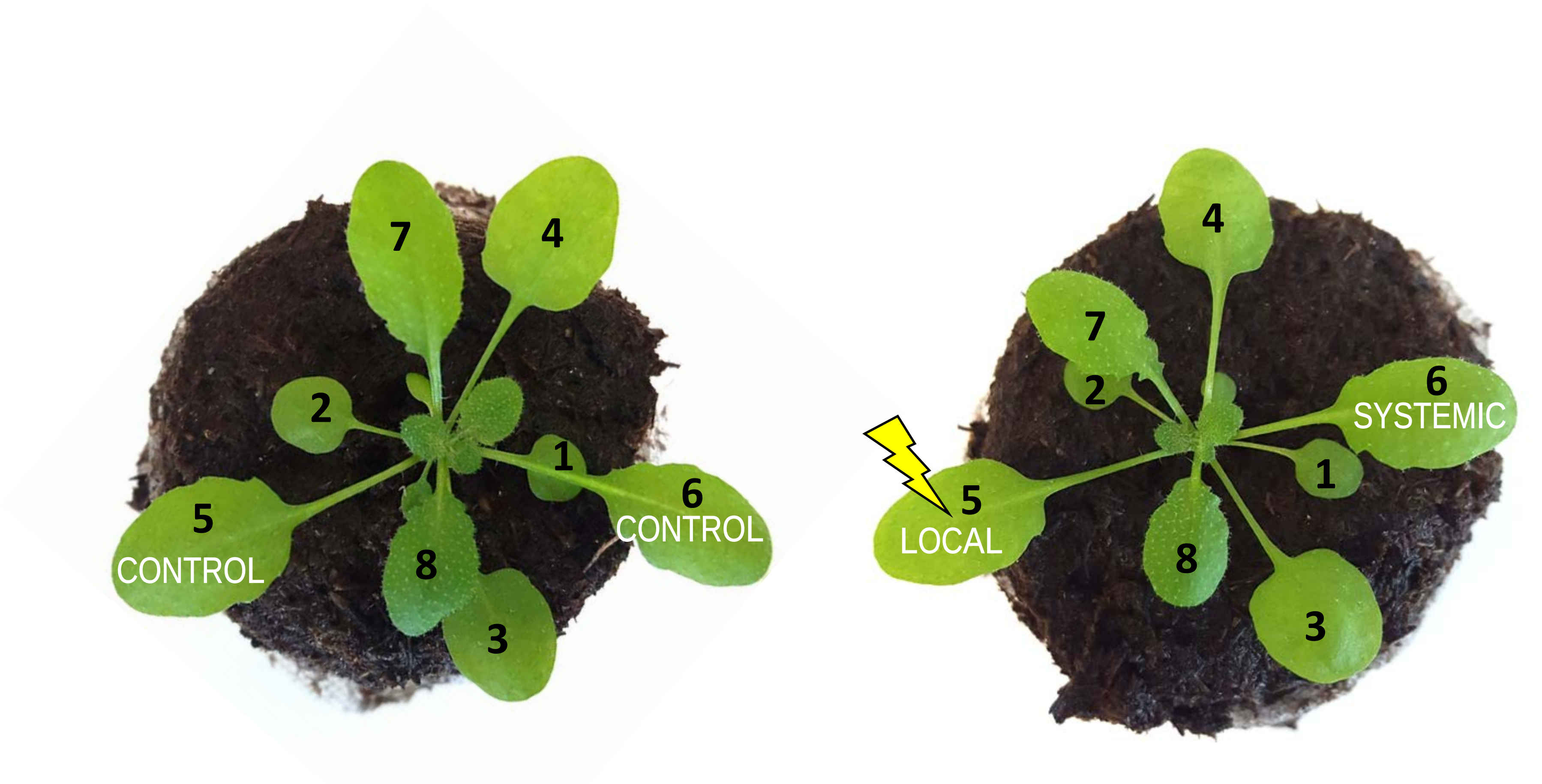

Fig. S8. Position of leaves used for electrolyte leakage assay. Leaves that were used for the ion leakage experiment shown in Figure 7 were fully-expanded leaves (positions 5 or 6 for "control", and 5 and 6 for "local" and "systemic", respectively). Significant differences in electrolyte leakage were not found between control leaves in positions 5 and 6. In addition, in control plants, we did not find significant differences in electrolyte leakage between control leaves and leaves in position 5 locally treated with high light for 10 min, reflecting that those leaves were not significantly damaged by the stress treatment and showing their potential capacity to deal with high light.
