# Supplementary material for: Identification and characterization of a core set of ROS wave‐associated transcripts involved in the systemic acquired acclimation response of Arabidopsis to excess light
Source: Plant J. 2019 Jan 30;98(1):126–41. doi: 10.1111/tpj.14205 (PMC6850305; doi:10.1111/tpj.14205)
Supplement: Supplementary file 3 [file TPJ-98-126-s003.docx]

**Supplemental Material**

**Table S1** – Transcripts significantly elevated in their expression in local leaves in response to light stress.

**Table S2** - Transcripts significantly elevated in their expression in systemic leaves in response to the light stress treatment applied to a local leaf.

**Table S3** - List of overlapping transcripts between the transcriptomics response of local and systemic leaves to light stress applied to a local leaf.

**Table S4** – Gene ontology of overlapping transcripts between the transcriptomics response of local and systemic leaves to light stress.

**Table S5** - Transcripts significantly elevated in their expression in local leaves of *rbohD* plants in response to light stress.

**Table S6** - Transcripts significantly elevated in their expression in systemic leaves of *rbohD* plants in response to the light stress treatment applied to a local leaf.

**Table S7** - List of overlapping transcripts between the transcriptomics response of local and systemic leaves of *rbohD* plants to light stress applied to a local leaf.

**Table S8** - Transcripts significantly elevated in their expression in systemic leaves of wild type plants, but not in systemic leaves of *rbohD* plants.

**Table S9** - Gene ontology of transcripts significantly elevated in their expression in systemic leaves of wild type plants, but not in systemic leaves of *rbohD* plants.

**Table S10** - Transcripts significantly elevated in their expression in seedlings in response to the external application of H_2_O_2_.

**Table S11** - List of *rbohD*-dependent systemic transcripts that are also significantly elevated in their expression in response to the external application of H_2_O_2_.

**Table S12** - List of transcripts suppressed in their local expression by DPI.

**Table S13** - List of transcripts suppressed in their systemic expression by DPI.

**Table S14** - Summary statistics reading for the sequencing performed.

**Table S15** – Summary hypergeometric testing for the different Venn diagrams.

**Fig. S1 -** Definition of local and systemic leaves used for this study.

**Fig. S2 -** Response of *Zat12::Luciferase* reporter plants to local application of light stress. Results are presented for 3 individual plants. Col plants expressing the Zat12::Luciferase reporter construct were grown and imaged with a NightOWL LB983 NC100 (Berthold, https://www.berthold.com/) imager as described in Devireddy et al., 2018.

**Fig. S3 -** Smear (Bland–Altman) plots generated in edgeR for local and systemic leaves at 0 and 2 min, and for the H_2_O_2_ treatment at 0 and 8 min. (a) Col Local Leaf (b) Col systemic leaf (c) rbohD local leaf, (d) rbohD systemic leaf, and (e) Seedlings exposed to H_2_O_2_.

**Fig. S4 -** Expression pattern of selected gene ontology groups in local and systemic leaves. See Fig. 2.

**Fig. S5 -** Heatmap representation of Figure 3. Fold-changes were log2 transformed, and used to generate the figure using the ComplexHeatmap package version 1.18.1 available in BioConductor 3.7. Rows were initially grouped by their transcription factor family, and then separately subjected to hierarchical clustering with complete linkage cluster analyses of their Euclidean distance.

**Fig. S6 -** Expression pattern (in counts) of the 7 transcripts selected for analysis using knockout mutants (Figure 7).

**Fig. S7 -** Expression pattern (in fold) of the 7 transcripts selected for analysis using knockout mutants (Figure 7).

**Fig. S8 -** Position of leaves used for electrolyte leakage assay. Leaves that were used for the ion leakage experiment shown in Figure 7 were fully-expanded leaves (positions 5 or 6 for “control”, and 5 and 6 for “local” and “systemic”, respectively). Significant differences in electrolyte leakage were not found between control leaves in positions 5 and 6. In addition, in control plants, we did not find significant differences in electrolyte leakage between control leaves and leaves in position 5 locally treated with high light for 10 min, reflecting that those leaves were not significantly damaged by the stress treatment and showing their potential capacity to deal with high light.
